# Supplementary material for: Usability of the REHOME Solution for the Telerehabilitation in Neurological Diseases: Preliminary Results on Motor and Cognitive Platforms
Source: Sensors (Basel). 2022 Dec 4;22(23):9467. doi: 10.3390/s22239467 (PMC9740672; doi:10.3390/s22239467)
Supplement: Supplementary file 1 [file sensors-22-09467-s001.zip › sensors-2028845-supplementary.pdf]

Supplementary Materials for "Usability of the REHOME solution for the telerehabilitation in neurological diseases: preliminary results on motor and cognitive platforms."

1. Patient satisfaction questionnaire for Spatial Memory Domain component on elderly people (CRGP platform)

Table S.1 reports the ad-hoc Patient Satisfaction Questionnaire (PSQ) used to assess the usability and user experience (motivation and satisfaction) for the spatial memory domain component of the CRGP platform. A 10-point Likert scale was proposed for each of the 6 items that were administered to participants to self-report their experience of using the videogame.

Table S1. Patient Satisfaction Questionnaire: items, questions, and response range.

Item	Question [a]	Response range
1	How often did you think about something else while using *MindTheCity!*?	Never (0) - Always (10)
2	How much did you feel rewarded during the training?	Very frustrated (0) - Very rewarded (10)
3	Did you find the videogame interesting?	Very boring (0) – Very interesting (10)
4	Was *MindTheCity!* fun to use?	Not fun at all (0) – Very fun (10)
5	Did you experienced anxiety while using the videogame?	Not at all (0) – Very much (10)
6	Was it easy to move inside the virtual environment?	Not at all (0) – Very much (10)

[a]: The questions were administered in Italian, as all recruited subjects were Italian. It was translated only for editorial purposes

2. Usability questionnaire for Assessment of motor condition on PD subjects (MREP platform)

Table S.2 reports the ad-hoc questionnaire used to evaluate usability of the assessment of motor condition component of the MREP platform.

Some open feedback sections were also included in which the user could explicitly provide explanations of the answers and perceived difficulties during the session: this allowed us to gather information and suggestions useful for improving the solution with a view to subsequent home experimentation.

Table S2. Usability questionnaire: items, questions, categories, and responses.

Item	Question [a]	Categories	Responses [c]
1	How often do you use your PC?	Technological skills	Never (0), Sometimes (1), Often (2)
2	Was your experience positive?	Overall Satisfaction	No (0), Partially (1), Yes (2)
3	Could the system be helpful for rehabilitation?	System Usefulness	No (0), Partially (1), Yes (2)

4	Could the system be a viable alternative to gym exercises?	System Usefulness	No (0), Partially (1), Yes (2)
5	Was the system clear and easy-to-use? (even without help)	System Usability	No (0), Partially (1), Yes (2)
6	Was the voice helpful?	System Usability	No (0), Partially (1), Yes (2)
7	Were the task instructions (text messages) easily readable?	System Usability	No (0), Partially (1), Yes (2)
8	What was the difficulty of traditional motor tasks (LA, AC, PoS, Gait)[b]	System Ease-of-use	Hard (0), Medium (1), Easy (2)
9	What was the difficulty of gamified motor tasks (LWL, FWL, BB)[b]	System Ease-of-use	Hard (0), Medium (1), Easy (2)
10	What was the engagement of gamified motor tasks (LWL, FWL, BB)[b]	User Engagement	Low (0), Medium (1), High (2)
11	Did you feel fatigued after the complete session?	User Perceived Status	Yes (0), Partially (1), No (2)
12	Did you have difficulty in using the system?	User Perceived Status	Yes (0), Partially (1), No (2)

[a]: The questions were administered in Italian, as all recruited subjects were Italian. It was translated only for editorial purposes
[b]: A response was provided for each motor task
[c]: Assigned points in the brackets were not visible to participants

3. Usability questionnaire for Motor Rehabilitation component on subjects with motor impairment (MREP platform)

Table S.3 reports the SUS questionnaire used to evaluate usability of the motor rehabilitation component (exergames in virtual environment) of the MREP platform.

Five responses, ranging from Strongly Disagree to Strongly Agree, were proposed for each item. Each response is assigned a score on a 5-point scale, as follows: Strongly Disagree=1 points, Disagree=2 points, Neutral=3 points, Agree=4 points, Strongly Agree=5 points. The maximum SUS score is 100 points, while the average score is 68 points. A SUS score above or below the average provides an immediate indication of overall usability perception. The SUS score is calculated using the following steps:

1. $SUM_{ODD} = (\sum_{odd} score\ odd\ items) - 5$
2. $SUM_{EVEN} = 25 - (\sum_{even} score\ even\ items)$
3. $SUS_{SCORE} = (SUM_{ODD} + SUM_{EVEN}) * 2.5$

Table S3. SUS questionnaire items, questions, and response range.

Item	Question [a]	Response range
1	I like to use this system more often.	Strongly Disagree (1) – Strongly Agree (5)
2	I find this system to be more complicated than it should be.	Strongly Disagree (1) – Strongly Agree (5)
3	I think the system is simple and easy to use.	Strongly Disagree (1) – Strongly Agree (5)
4	I need technical support to use this system.	Strongly Disagree (1) – Strongly Agree (5)
5	I find the system functioning smoothly and is well integrated.	Strongly Disagree (1) – Strongly Agree (5)

6	I think there are a lot of irregularities in the system?	Strongly Disagree (1) – Strongly Agree (5)
7	I think most people can learn this system quickly.	Strongly Disagree (1) – Strongly Agree (5)
8	I find this system to be time-consuming.	Strongly Disagree (1) – Strongly Agree (5)
9	I feel confident while using this system.	Strongly Disagree (1) – Strongly Agree (5)
10	I think there are a lot of things to learn before I can start using this system.	Strongly Disagree (1) – Strongly Agree (5)

[a]: The questions were administered in Italian, as all recruited subjects were Italian. It was translated only for editorial purposes
